# Syphilization

**Published:** 1865-10

**Authors:** 


### SYPHILIZATION.

Prof. W. Boeck, of Christiana, delivered at the theatre of the Meath Hospital, before the principal physicians and surgeons of Dublin, the following lecture on this subject:

By syphilization I understand the mode of treatment by which, by repeated inoculations of syphilitic matter, taken from primary sores, I bring the body into the condition that it is no longer susceptible of the action of the syphilitic virus.

It will, perhaps, be agreeable to you, gentlemen, before I proceed further, that I should lay before you a short *resume* of the history of this mode of treatment. Auzias-Turenne, of Paris, performed inoculations of syphilitic matter upon animals in order to see whether this virus could be transferred to them, which up to that time had been denied. In this he was at length successful, and it was chiefly apes which could with the greatest facility be inoculated. After chancres had been repeatedly produced in the same ape, a great many sceptical physicians wished to see his inoculation, and a meeting was appointed in the *Jardin des Plantes;* the old ape was inoculated, and a still greater crowd assembled a few days later to see the result. But when the ape was brought in nothing was to be seen. It may easily be imagined how the result was received, and that Auzias-Turenne was ridiculed, but he did not on that account give up the method; he continued his inoculations, found that the old ape was not susceptible of fresh inoculations, but that a second ape after inoculations got chancres, though this ape also after a series of inoculations became unsusceptible.

Auzias-Turenne now saw clearly that he had here a natural law, in itself resembling that which your immortal Jenner had discovered in the inoculation of vaccine matter, and we shall not upbraid him that his French blood now carried him away, and that his first idea was to employ the inoculation of syphilitic matter like that of vaccine matter—as a prophylactic. We cannot gainsay him that his train of ideas is logically correct, but it is not practically correct, for the great rule is, that he only gets syphilis who himself will have it.

As the result of this idea of employing syphilization as a prophylactic, my friend Auzias wished at the time to syphilize all public girls, seamen, and soldiers, and he would willingly have syphilized us all. No wonder, then, that such an idea met with all the opposition it deserved; but it was not long until Auzias renounced his error, and at the same time there appeared an Italian, Sperino, of Turin, who showed, by a series of experiments, that the syphilitic disease was cured during these inoculations, which Auzias, too, at the same time, demonstrated. Still, this failed to reconcile physicians to the new method; such a prejudice had been raised against it that both the Académie de Médecine of Paris and the Academy of Turin condemned it without having the necessary materials before them for passing any judgment; the paradox involved in this method appeared to all so enormous as to render proofs of its absurdity unnecessary.

Lecturing in the University of Christiania upon syphilis, and having a section of the hospital devoted to this disease, I carefully investigated all that was advanced upon this subject, and ascertained that there must be some truth in it. I had, through a period of very many years, found that our treatment with mercury is highly unsatisfactory; I therefore considered that, from my position, it was my duty to give a trial to this new method, although it appeared to me as paradoxical as it did to all the world, and notwithstanding that it had been condemned by two academies. But before I began, I laid down for myself certain limits, to which I still adhere. It will be at once observed that I will not speak of the method as a prophylactic; this would be immoral; but neither am I at liberty to employ it in every case of syphilis; it is only when syphilis has become constitutional—when the syphilitic virus flows with every drop of blood through the system—that I allow myself also to inoculate it upon the skin.

The next question is, whether I shall employ syphilization in every case of constitutional syphilis?

By a fortunate coincidence it happened that of the two individuals whom I first took under treatment by syphilization, the one had not been treated for syphilis, while the other had been the subject of all the resources of our art. In the first the inoculations proceeded without difficulty, the symptoms gradually disappeared—in a word, I found myself upon the beaten path. In the other case all was irregular, I could effect no order at all, and when my first patient was well, the phenomena in the second were still in full bloom. I immediately began to suspect that it was to the medicines previously given that this result was attributable, and on subsequently investigating this opinion, its truth has been most completely confirmed; so that I have made it a general rule to syphilize only those who have not previously been treated with mercury, whether this has been employed for primary or constitutional symptoms. But if I be asked whether syphilization has not some effect in these cases, I can answer decidedly in the affirmative—it often acts incredibly. Dr. Simpson, of Edinburgh, has recently described two such cases, which were sent over to me by Professor Simpson; what is there stated corresponds precisely to what I have myself noted, and of which any one may satisfy himself. But the reason why I do not undertake the treatment of such individuals is to avoid having relapses, which in these cases are apt to occur.

Now, in order to make my usual mode of proceeding as plain as possible, I shall suppose that a person laboring under primary syphilis consults me. In this case I treat the primary sore as a simple ulcer—I prescribe a weak solution of sulphate of zinc or such like, and occasionally employ a slight cauterization with nitrate of silver; I give no internal medicine, but make the patient come to me once or twice a week, that I may observe when the constitutional symptoms break out, for the earlier syphilization can be commenced the better. So soon as I perceive the first constitutional signs, I commence the treatment by taking matter from an indurated chancre or from an artificial pustule in a patient under treatment by syphilization. I inoculate first on both sides of the chest, and make three punctures with a lancet, precisely in the mode adopted in vaccinating. After three days pustules are developed, and then I inoculate again in the sides, taking the matter from the pustules produced by the first inoculation, observing carefully to make the second inoculation at a distance from the first, so that the sores may not become confluent. At the end of three days I make the third inoculation, taking the matter from the pustules of the second inoculation; and I now continue to inoculate on both sides every third day, always taking the matter for the fresh inoculation from the pustules last formed, so long as this matter continues to afford a positive result. When it no longer takes, I procure new matter in the same mode as for the first inoculation, and continue with this as with the first. This second matter will yield smaller sores and a shorter series than the first, and when it no longer takes I procure a third and proceed in the same manner. This third matter will produce very little effect, and I therefore pass to the upper arm, where I proceed in precisely the same mode as in the sides; and when no effect is any longer visible in the upper arm I remove to the thighs, and continue there in the same way as in the two preceding places. By the time the inoculations are here brought to an end, from three to three and a half or four months have probably elapsed, the symptoms which manifested themselves from the commencement have disappeared, or if some slight symptom has remained this disappears spontaneously. It often happens that during the treatment a fresh outbreak takes place, and he who is not scquainted with the method believes that some other plan must now be adopted; another infers that syphilization is of no avail. But, let them not be deterred by any symptom, not even by the most severe iritis, which never requires anything but the instillation of a little atropia. But, happen what may, let them shut their eyes to it and continue the inoculations. The patient who, during the whole treatment, can attend to his business, feels, after it is completed, perfectly well, and may immediately expose himself to any hardships. He can endure wet, cold—in a word, everything which after mercurial treatment would render him liable to life-long illness. It is probable that I may now be asked as to the result at a later period for these individuals, and I shall speak first of the relapses. On the whole, I have treated 429 individuals, and of these 45 have come back, making about 10½ per cent.; but, as we may calculate that some of those treated during the last year will return, I will assume that the relapses will amount to 12 or 13 per cent. But, let us now examine more closely what is called a relapse after syphilization. In many instances a single mucous tubercle, a small white spot on the tongue or in the throat—symptoms for which nothing more than external means is employed, and for which the patients are treated only for a few days in hospital. So far as I at this moment remember, thirteen were taken again under treatment with syphilization, and two with iodide of potassium.

You will next ask whether tertiary symptoms have been developed in any of them. This has been the case, I believe, with three; but at the same time these individuals have been perfectly well—their general health has not, as so often happens after mercurial treatment, been broken down, and in those who have had relapses it has been good, as it is evident that in those who have had no relapse it has been particularly good.

We come now to the children of those who have been syphilized. Here we are not much better off than after the mercurial treatment; we see the same rule to prevail as after this last method, namely—that when the mother has been syphilitic, the first child or children is or are syphilitic; that they are healthy is the exception. If the father has been syphilitic, the children are, in general, healthy; that they are syphilitic is the exception.

You will next propose to me the question how I treat syphilitic children. I treat them precisely as I do adults; and it is interesting to see that the sores in these little ones bear in size a proportion to that of the child, and that the patients suffer less, and not more than adults. The results of syphilization in children with hereditary syphilis have not been brilliant; of forty-two children, twenty-two died, but I have taken under treatment every case that I have met with, and every one knows that in such children there are very often affections of the internal organs which lie beyond our power to cure. I cannot at this moment say how many little children with acquired syphilized, but they are not few, and of these only one died, the cause of death in that instance being croup after I had performed tracheotomy. Of adults, two died—an old woman of dysentery, and a young women of puerperal fever. This latter case I forget to include in the *resume* I have given in the *British Medical Journal.*

Now, in order to give you a definite idea of the confidence I have in this method after having practised it daily for thirteen years, I shall say only that if I myself, or any of mine, were so unfortunate as to get syphilis, I should employ no other means than syphilization.

Still a few words in conclusion, gentlemen. Vaccination has for many years stood alone; syphilization now comes to join it. Shall we stop here? I believe not. Vaccine and the syphilitic matter are both animal viruses; we see them contained under a similar law. May not also the other animal poisons be referred to a similar law? We see that nature is simple in her diversity: should this not also here be the case? Should not glanders, hydrophobia. etc., some time be curable? Let us all seek to clear up this dark point in our science, and let us not, as hitherto, with respect to syphilization, seek only to extinguish the rising gleam.—*Med. Times and Gaz.*, June 10, 1865. *American Medical Journal.*

